# Fasudil Protects the Heart against Ischemia-Reperfusion Injury by Attenuating Endoplasmic Reticulum Stress and Modulating SERCA Activity: The Differential Role for PI3K/Akt and JAK2/STAT3 Signaling Pathways

**DOI:** 10.1371/journal.pone.0048115

**Published:** 2012-10-31

**Authors:** Yapeng Li, Wei Zhu, Jianping Tao, Ping Xin, Mingya Liu, Jingbo Li, Meng Wei

**Affiliations:** Division of Cardiology, Shanghai Sixth Hospital, Shanghai Jiao Tong University School of Medicine, State Key Discipline Division, Shanghai, People's of Republic of China; University of Pecs Medical School, Hungary

## Abstract

Disordered calcium homeostasis can lead to endoplasmic reticulum (ER) stress. Our previous data showed that time course activation of ER stress contributes to time-related increase in ischemia-reperfusion (I/R) injury. However, it has not been tested whether PI3K/Akt and JAK2/STAT3 pathways play differential roles in reducing ER stress to protect the heart. In the present study, using fasudil which is a specific inhibitor of ROCK, we aimed to investigate whether improved SERCA expression and activity accounts for reduced ER stress by ROCK inhibition, specifically whether PI3K/Akt and JAK2/STAT3 pathways are differentially involved in modulating SERCA activity to reduce ER stress and hence I/R injury. The results showed that during the reperfusion period following 45 min of coronary ligation the infarct size (IS) increased from 3 h of reperfusion (45.4±5.57%) to 24 h reperfusion (64.21±5.43, P<0.05), which was associated with ER stress dependent apoptosis signaling activation including CHOP, Caspase-12 and JNK (P<0.05, respectively).The dynamic ER stress activation was also related to impaired SERCA activity at 24 h of reperfusion. Administration of fasudil at 10 mg/Kg significantly attenuated ROCK activation during reperfusion and resulted in an improved SERCA activity which was closely associated with decreases in temporal activation of ER stress and IS changes. Interestingly, while both PI3K/Akt and JAK2/STAT3 signaling pathways played equal role in the protection offered by ROCK inhibition at 3 h of reperfusion, the rescued SERCA expression and activity at 24 h of reperfusion by fasudil was mainly due to JAK2/STAT3 activation, in which PI3K/Akt signaling shared much less roles.

## Introduction

Timely effective reperfusion therapy remains the main therapeutic strategy for treating acute myocardial infarction, however, the beneficial effects can be compromised by ischemia/reperfusion (I/R) injury [1], [2]. Different measures have been developed to protect against I/R injury, including various strategies of ischemic pre- or post-conditioning. However, the efficacy and/or efficiency of these interventions have been questioned, which becomes a barrier for its clinical application. This is largely because the pathological process of I/R injury is complicated and one simple mechanism via which the existing intervention protects the heart certainly is not sufficient to prevent and/or reverse the damage caused by I/R injury. The fact that the role of the classical “reperfusion injury survival kinase (RISK) pathway” in cardiac protection [3] is being challenged by the novel “survivor activating factor enhancement (SAFE) pathway” [4] also indicates complicated pathological processes involved in I/R injury. Further study is certainly warranted to elucidate the different roles for these two signaling pathways.

Recent studies demonstrated that endoplasmic reticulum (ER) stress is also involved in pathological I/R injury process [5], [6], [7]. ER stress refers to a condition in which normal ER function is impaired, leading to accumulation of unfolded or mis-folded proteins in the ER lumen [8]. In the case of severe or prolonged ER stress, the endogenous ER stress responses cannot relieve the cell from the stress and result in the activation of ER stress related apoptosis signaling pathways, including C/EBP homologous protein (CHOP)-, c-Jun NH2-terminal kinase (JNK)-, and caspase-12 dependent pathways [9]. Previous studies showed that impaired activity of sarco/endoplasmic reticulum calcium ATPase (SERCA), a Ca^2+^ ATPase that transports Ca^2+^ from the cytosol of the cell to the lumen of the sarcoplasmic reticulum, can induce ER stress [10], [11], whereas well preserved SERCA activity attenuates ER stress and hence protects against myocardial I/R injury [12], [13]. It would be interesting to test whether PI3K/Akt and/or Janus kinase (JAK)2/Signal transducer and activator of transcription (STAT)3 can modulate SERCA activity to protect against ER stress.

Importantly, in line with previous studies [14], [15], [16], our recent study demonstrates that I/R injury exhibited a time-course of changes during the first 24 hours of reperfusion. We showed that in an in *vivo* rat I/R injury model induced by 30 min of coronary ligation followed by reperfusion, infarct size (IS) increased at 24 h of reperfusion compared with at 2 h of reperfusion, and this temporal increase in I/R injury was associated with dynamic ER stress activation. Interestingly, apelin infusion can attenuate this time-related increase in IS, which was closely associated with an improved ER stress [17].

Studies have shown that Rho-kinase (ROCK) is involved in various basic cellular biological activities, and the beneficial effects of ROCK inhibition against I/R injury by fasudil have been well demonstrated [18], [19], [20]. Therefore, in the present study using an *in vivo* rat heart I/R injury model we aimed to investigate whether ROCK inhibition by fasudil can attenuate the time- related increase in I/R injury through reduced ER stress activation for which PI3K/Akt and JAK2/STAT3 pathways might play differential roles, specifically whether SERCA activity is differentially modulated by these two signaling pathways during the reperfusion period.

## Materials and Methods

### Animals

Eight week-old male Sprague-Dawley rats, weighing between 250 g and 280 g (Experimental Animal Center, Fudan University, Shanghai, P. R. China) were studied. The animal research study protocol was in compliance with ‘The Guide for the Care of Use of Laboratory Animals’ published by the National Institute of Health (NIH Publication No. 85-23, revised 1996) and approved by the Animal Care Committee of Shanghai Sixth Hospital, Shanghai Jiao Tong University School of Medicine. All the rats were housed for two weeks for an acclimatization period before the experiments.

### *In vivo* heart I/R injury model

The rat heart I/R injury model has been established in our laboratory and described previously [21]. Briefly, after anesthesia with sodium pentobarbital (50 mg/kg, IP), rats were intubated and ventilated with room air and body temperature was maintained at 37°C using a heating pad and monitored with a thermometer. Using a sterile surgical approach, the right carotid artery and vein were cannulated with a 24-gauge angiocatheter for drug infusion. Left thoracotomy was also performed and the heart was exposed. After pericardiotomy, the left coronary artery (LCA) was encircled by a 6-0 prolene suture just distal to its first branch and its ends were threaded through a polyethylene tubing (PE-50) to form a snare for reversible coronary artery occlusion. Prior to LCA occlusion, the animals were anti-coagulated (150 U/Kg sodium heparin). Cardiac ischemia was confirmed by an initially pale and later cyanotic area below the suture and ST-T elevation shown in ECG, while reperfusion was characterized by rapid disappearance of cyanosis followed by vascular blush. Ischemia duration was 45 minutes and hearts were examined at 3, 12 and 24 hours after reperfusion to assess the temporal changes in I/R injury.

### Area at risk and IS determination

After the animals were sacrificed, the LCA was re-occluded and 2% Evans blue dye was retrogradely injected into the ascending aorta to delineate the area at risk. The heart was then cut into 5 to 6 transverse slices and incubated for 15 min at 37°C in a phosphate-buffered 1% 2,3,5-triphenyltetrazolium chloride solution to determine infarcted myocardium. The extent of the area of necrosis was quantified by computerized planimetry and corrected for the weight of the tissue slices. IS was expressed as percentage of total weight of LV at risk area [21].

### Western blotting

Western blot was performed on heart tissue obtained from area at risk. After homogenization and protein quantification, 40 ug of protein from each sample was separated in 10 to 12% Tris-glycine SDS gels by electrophoresis and then transferred to polyvinylidene difluoride (PVDF) membranes. After blocking with 5% BSA in Tris-buffer, membranes were incubated with the primary antibodies. Primary antibodies targeting ezrin/radixin/moesin (ERM), p-Ezrin (Thr567)/Radixin(Thr564)/Moesin(Thr558), JNK, p-JNK (Thr183/Tyr185), Akt, p-Akt (Ser473), p-JAK2 (Tyr1007/1008), STAT3 and p-STAT3 (Ser727) were obtained from Cell Signaling Technology; 78 kD glucose-regulated protein (GRP78), Caspase-12 form Sigma-Aldrich; CHOP, phospholamban (PLB), p-PLB (Ser16), JAK2, SERCA2, and β-actin were purchased from Santa Cruz Biotechnology, SERCA2a from Affinity Bioreagent and SERCA2b from Calbiochem. HRP-conjugated secondary antibody was then used. ECL western blotting detection kit (Pierce) was finally used to measure immunoreaction with a light sensitive film (Kodak, USA). The band for each protein was then quantified by densitometry using Image J software (version 1.41, NIH, USA) and normalized to the expression of β-actin for protein loading.

### Evaluation of apoptosis activation

Using a commercially available kit (Roche, Germany), TUNEL staining was performed the same way as reported previously [21]. For each heart tissue, at least 100 cells from peri-infarct area were counted in 10 fields (×400 magnification, Leica Q500MC, Germany). The percentage of TUNEL staining positive cells was calculated as follows: number of apoptotic cells/total number counted ×100%.

Similar to the method described previously [22], Caspase-3 activity was measured using a commercial available kit (CASP3C, Sigma) for heart tissue based on amount of p-nitoanilide cleaved from peptide (acetyl-Asp-Glu-Val-Asp p-nitoanilide) by caspase-3 per minutes which can be determined from the absorbance value at 405 nm. All the samples were expressed as percentage increase in comparison with controls.

### SERCA activity quantification

Using a commercially available kit (Nanjing Jiancheng Bioengineering Institute, Nanjing, China) SERCA activity was measured based on the inorganic phosphate produced [23]. Microsomal fraction was obtained using a commercially available isolation kit (ER isolation kit, #ER0100, Sigma Aldrich), heart tissue at risk area (500 mg) was collected and washed twice with PBS solution and then cut into small pieces. Isotonic extraction buffer from ER isolation kit (5 mL, 1×) was added. The sample was homogenized in a glass tube homogenizer and ER fractions were isolated according to the manufacturer's protocol. After ultracentrifuging (Beckman Coulter Optima TLX-120) to obtain ER fraction, the pellet was resuspended in 500 µL of 1× isotonic extraction buffer and homogenized in a glass tube homogenizer. Protein concentration of the microsomal fraction was quantified and the ATPase activities were determined using the same method as previously described [24].

### Pharmacological interventions

To test whether ROCK inhibition can protect against ER stress, fasudil, a specific antagonist for ROCK activity (10 mg/kg, Tianjin Chase Sun Pharmaceutical Co. Ltd, Tianjin, P. R. China) was given intravenously as a bolus at 15 minutes before reperfusion. Pharmacological blockers, including LY294002 (PI3K/Akt inhibitor, 0.3 mg/kg; Calbiochem) and AG490 (JAK2 inhibitor, 3 mg/kg; Sigma Aldrich) were dissolved in DMSO (<300 ug/kg) and were given intravenously as a bolus at 10 minutes before LCA occlusion to test the role for each specific signaling pathway. To test whether modulation of ER stress itself could decrease the dynamic changes in I/R caused by temporal activation of ER stress, tauroursodeoxycholic acid (TUDCA), a chemical chaperone which can regulate ER stress [25], was also used and administered right before ischemia, thus the role of ER stress in I/R injury was further evaluated. In addition, different doses of TUDCA (100, 200 and 400 mg/Kg, IP) based on the previous literature [26] were also tested to describe the dose-dependent effects of TUDCA on regulating ER stress to protect against I/R injury during reperfusion. Finally,Y-27632 (Calbiochem, USA), another pharmacological inhibitor of ROCK which is structurally different from fasudil, was also orally administered (30 mg/Kg by gavage) before anesthesia to test if different effects could be obtained and thus to exclude any potential off-target effects of fasudil [27].

### Statistical analysis

All the data are expressed as mean ± SD. One-way ANOVA followed by post hoc analysis using Student-Newman-Keuls test were used for statistical analysis. A P value of less than 0.05 was considered as statistically significant. Statistical analyses were performed using SPSS 13.0 statistics software (SPSS Inc., Chicago, IL, USA).

## Results

### ROCK Inhibition attenuated I/R injury through inhibition of time-related ER stress dependent apoptosis

**Figure 1:**
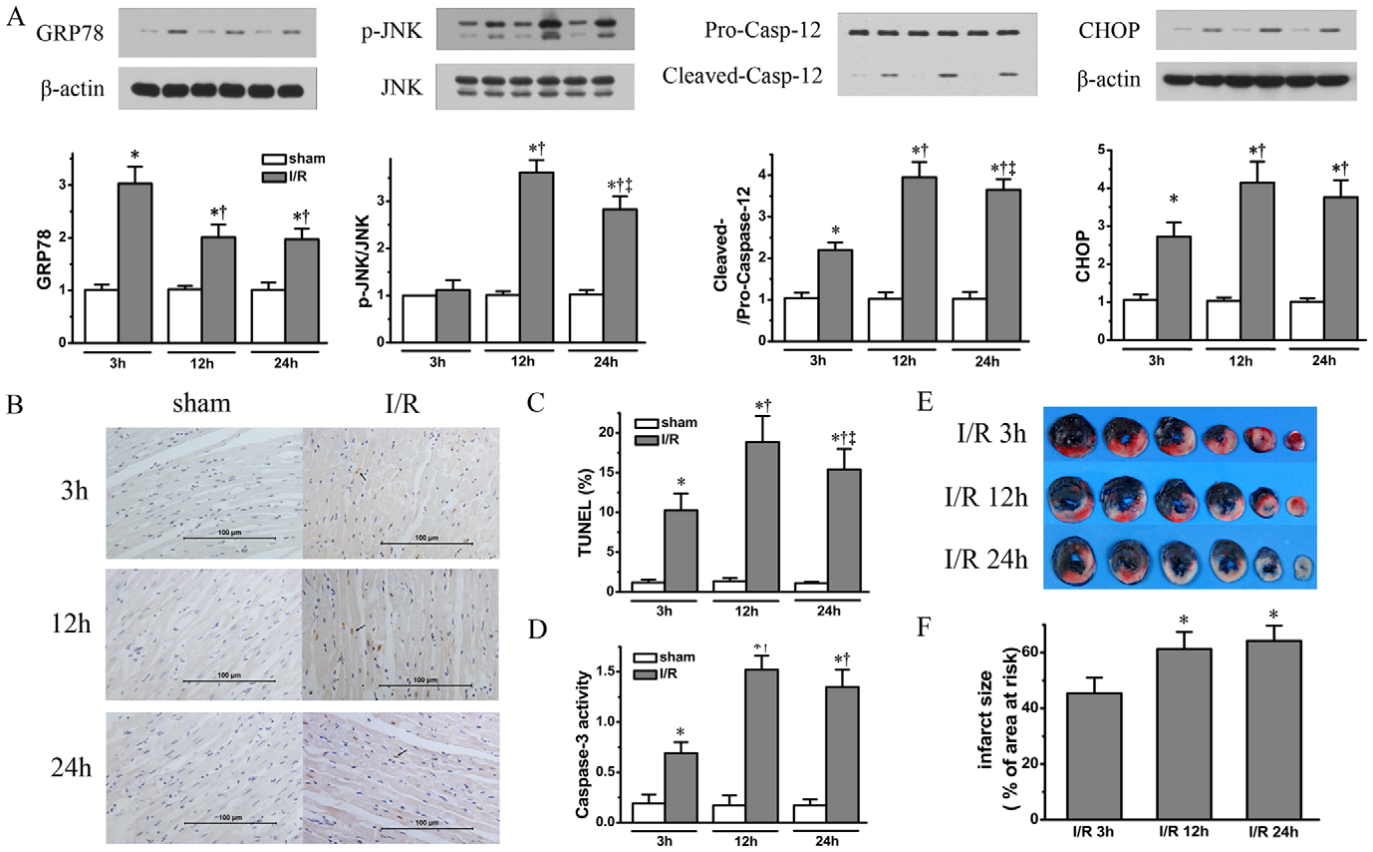
Time course increases in I/R injury associated with dynamic changes in ER stress signalings. In panel A, ER stress related protein markers including GRP78, phosphorylation level of JNK, cleaved Caspase-12 band and CHOP were quantified for IR rats in comparison with Sham operated rats. Apoptosis signaling was assessed by TUNEL staining using paraffin embedded heart tissue (Panel B) with bar graph showing percentage TUNEL staining positive cells (Panel C). Caspase-3 activity was also quantified (Panel D) (n = 6 for I/R group and n = 4 for sham group at each time point). Representative sequential LV slices obtained from each group in panel E show both area at risk demarcated with Evans blue staining for normal heart tissue and area of necrosis with 2,3,5-triphenyltetrazolium chloride staining (pale area = infarcted tissue). The bar graph shows the percentage of LV weight of infarct area over LV weight at area at risk (Panel F), demonstrating time course changes in infarct size at 3, 12 and 24 hours of reperfusion following 45 minutes ischemia (n = 8 for each time point/group). All the data presented in the bar graphs were expressed as mean±SD. * denotes P<0.05 vs. Sham groups; †, P<0.05 vs. I/R group at 3 hours of reperfusion and ‡, P<0.05 vs. I/R group at 12 hours of reperfusion.

Consistent with our previous study [17], we observed a similar pattern of dynamic changes in ER stress related apoptosis signaling during the 24 h period after 45 min of LAD ligation, with p-JNK, cleaved Caspase-12 and CHOP exhibiting a peak in their protein expression at 12 h of reperfusion (*P*<0.05, vs. at 3 h, respectively) followed by a decrease at 24 h (*P*<0.05, vs. at 12 h, respectively) which was, however, still higher than at 3 h of reperfusion (*P*<0.05, vs. at 3 h, respectively). Interestingly, the level of CHOP expression at 24 h of reperfusion was not significantly down-regulated compared with at 12 h (*P*>0.05), and p-JNK did not show an increase at 3 h of reperfusion. In contrast, GRP78 had a peak at 3 h of reperfusion (*P*<0.05, respectively) which was then decreased at 12 h and 24 h of reperfusion (*P*<0.05, vs. at 3 h, respectively), yet remained at a higher level than sham controls (*P*<0.05, respectively) (Fig. 1A. n = 4 for sham group and n = 6 for I/R group at each time point).

In line with the ER stress related apoptosis signaling, percentage of TUNEL staining positive cells exhibited a similar pattern of changes, showing a significant increase from 3 h to 12 h of reperfusion (10.26±2.13% at 3 h vs. 18.85±3.28% at 12 h. *P*<0.05, respectively), followed a slight decrease at 24 h (15.41±2.58%. *P*<0.05 vs. 12 hours of reperfusion) which was still higher than at 3 h of reperfusion (*P*<0.05). The same was true for Caspase-3 activity quantification (Fig. 1B–D, the rats studied are the same as shown in Fig. 1A). Importantly, the IS increased significantly at 12 h (61.33±6.02%) compared with at 3 h (45.4±5.57%, *P*<0.05) after reperfusion, however, showed no further significant increase at 24 h (64.21±5.43%, *P*>0.05) (Fig. 1E&F, n = 8 for each time point).

**Figure 2:**
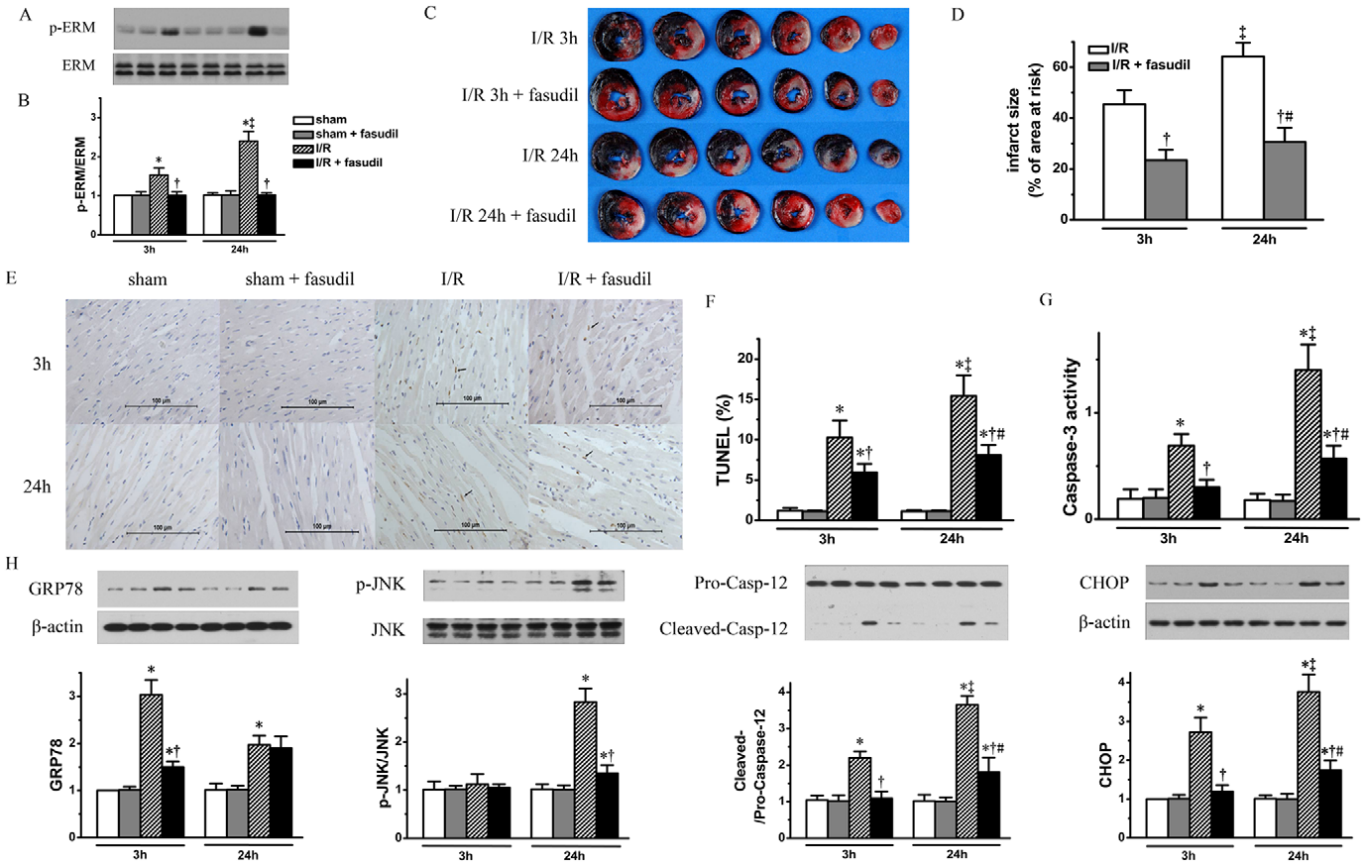
ROCK inhibition results in decreases in both I/R injury and ER stress activation. Representative bands of phosphorylated and total ERM (Panel A) with quantification shown in bar graph (Panel B) demonstrating that ERM was activated during I/R injury process and can be suppressed by fasudil infusion (n = 4 for sham operated and 6 for I/R group rats with or without fasudil therapy for each time point). IS was measured for I/R rats receiving placebo or fasudil therapy at both 3 and 24 hours (n = 8 for each group at each time point) after reperfusion with representative sequential heart slices shown in Panel C and IS quantification shown in bar graph (Panel D). Using the same rat heart samples as shown in Panel A, paraffin embedded heart tissue slices from LV papillary muscle level, TUNEL staining (Panel E) was perform with percentage of TUNEL staining positive cells quantification shown in bar graph (Panel F). Using heart tissue from risk area Caspase-3 activity (Panel G) was measured to quantify apoptosis. ER stress related apoptosis signaling was also quantified by measuring its protein markers including GRP78, p-JNK, cleaved Caspase-12 and CHOP, with representative bands quantified in corresponding bar graph (Panel H). All data expressed as mean±SD. * denotes P<0.05 vs. Sham groups; †, P<0.05 vs. I/R group at the same time point; ‡, P<0.05 vs. I/R group at 3 hours of reperfusion and #, P<0.05 vs. I/R+fasudil group at 3 hours of reperfusion.

After reperfusion, the ROCK activity, as assessed by phosphorylation levels of downstream target ERM (ezrin, radixin and moesin), was up-regulated with its activity being increased at 3 h (1.5 folds, *P*<0.05 vs. sham controls) and further enhanced at 24 h of reperfusion (2.4 folds, *P*<0.05 vs. 3 h). Administration of fasudil (10 mg/kg) before reperfusion completely abolished ROCK activation at both 3 h and 24 h of reperfusion (*P*<0.05, respectively) (Fig. 2 A&B, n = 4 for sham operated rats and 6 for I/R group rats treated with fasudil at each time point, data for the sham operated or I/R group rats without fasudil therapy are the same as in Fig. 1).

Inhibition of ROCK activation resulted in a reduction in IS not only at 3 h (23.41±4.11% vs. I/R group, 45.4±5.57%; *P*<0.05), but also at 24 h after reperfusion (30.57±5.63% vs. I/R group, 64.21±5.43%; *P*<0.05). More importantly, the time difference in IS between 3 h and 24 h (3 h, 45.4±5.57% to 24 h, 64.21±5.43%) within I/R group was significantly attenuated by fasudil administration from 19.23±4.89% to 7.15±4.93% (*P*<0.05. Fig. 2 C&D) (n = 8 for IS quantification for each group rats treated with fasudil at each time point, the other rats are the same as in Fig. 1E&F).

The apoptosis signaling pathways in I/R group, as shown in both percentage of TUNEL staining positive cells and Caspase-3 activity, were inhibited at the two time points by fasudil infusion (*P*<0.05 vs. I/R group, at 3 and 24 h, respectively), although higher apoptosis activities were still observed in fasudil treated I/R group rats at 24 h than at 3 h of reperfusion (*P*<0.05, respectively) (Fig. 2E–G, the rats studied are the same as in Fig. 2A).

Meanwhile, fasudil decreased GRP78 expression in I/R group rats at 3 h of reperfusion (*P*<0.05, vs. I/R group), without affecting its level at 24 h of reperfusion (Fig. 2H). Importantly, fasudil also significantly inhibited ER stress related apoptotic signaling as evidenced by decreases in p-JNK, cleaved Caspase-12 and CHOP expression levels, while having no effects in sham controls (Fig. 2H). However, the time related differences in expression levels between 3 and 24 h of reperfusion were still present for both cleaved Caspase-12 (*P*<0.05) and CHOP (*P*<0.05) in fasudil treated I/R group rats (Fig. 2H, the rats studied are the same as in Fig. 2A).

To further evaluate the causative effects of temporal activation of ER stress on dynamic changes in I/R injury during reperfusion, we have tested whether chemical chaperone TUDCA can exert time-dependent effects on reduction in IS. At 3 h of reperfusion, TUDCA resulted in a slight decrease in IS only when delivered at 400 mg/Kg (IS, 35.28±4.67%. P<0.05, vs. I/R group), with no significant changes at 100 mg/Kg (IS, 40.23±5.15%) and 200 mg/Kg (IS, 38.95±5.08%) compare with I/R group ( P>0.05. respectively). However, at 24 h of reperfusion, TUDCA dose-dependently suppressed the temporal increases in IS, showing significant reduction in IS at dose of 100 mg/Kg (50.21±5.38%) and 200 mg/Kg (48.53±5.64%) compared with I/R group, however, with no differences between these two doses (P>0.05). A further decrease in IS was achieved when TUDCA was delivered at 400 mg/Kg (41.43±4.73%. P<0.05, vs. at 100 or 200 mg/Kg, respectively). In contrast, Y-27632 as another ROCK inhibitor can exhibit its protection early at 3 h of reperfusion, resulting in an even lower IS (25.63±4.25%) than TUDCA given at 400 mg/Kg (P<0.05). Interestingly, at 24 h of reperfusion, even though Y-27632 showed a trend of further decrease in IS (32.72±4.59%) compared with TUDCA at 400 mg/Kg, which, however, did not reach statistical significance (Fig. S1), indicating equal effects at this late phase of reperfusion offered by these two pharmacological interventions (Fig. S1). ER stress related signalings including GRP78 and CHOP expressions were quantified at both 3 and 24 h of reperfusion for each dose TUDCA group and Y-27632, respectively (Fig. S2). At 3 h of reperfusion, TUDCA administered at 100 mg/Kg can decrease the up-regulated GRP78 expression (P<0.05), and when delivered at higher doses (200 and 400 mg/Kg) resulted in a further reduction in GRP78 expressions (P<0.05, vs. at 100 mg/Kg, respectively), yet without showing any differences compared with Y-27632 group (P>0.05). The same pattern of changes was observed in CHOP expressions at 3 h of reperfusion for the different interventions. In contrast, at 24 h of reperfusion, the differences of GRP78 expression between the different dose TUDCA groups were not present, showing similar expression levels as Y-27632 treated group. Paradoxically, at this late phase (24 h) of reperfusion, TUDCA at 100 mg/Kg still exhibited less effect on suppressing the CHOP expressions compared with at higher dose TUDCA groups which were not different from Y-27632 treated group (Fig. S2).

### Improved SERCA activity by fasudil through p-PLB and SERCA2 expression level

**Figure 3:**
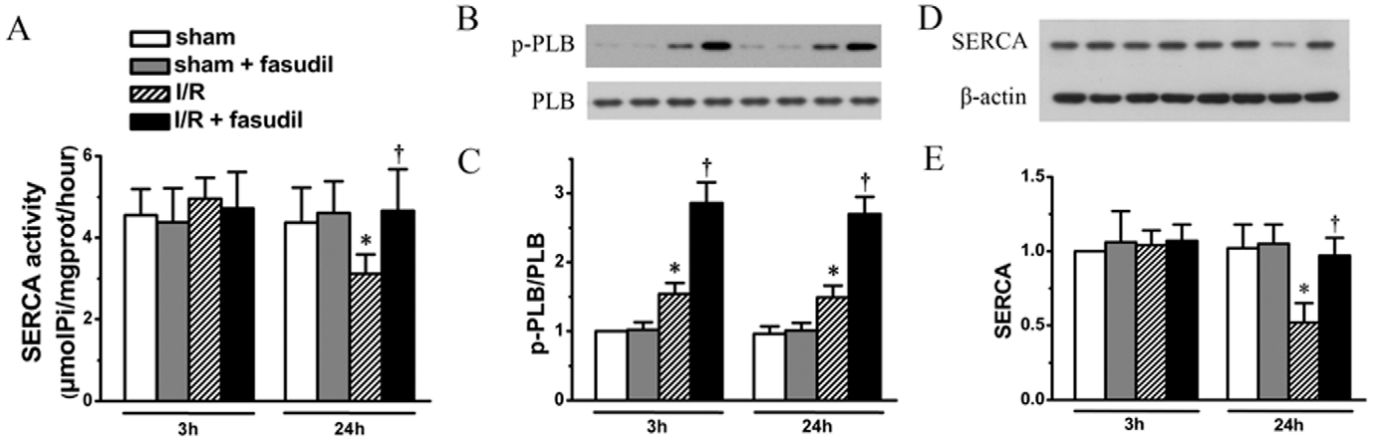
SERCA activity, PLB phosphorylation and SERCA protein expression levels were measured. Using the same tissue obtained from each group as described in figure 2, SERCA activity (Panel A), phosphorylation level of PLB (representative protein bands shown in Panel B and quantification in bar graph of Panel C) and SERCA protein expression level (Panel D & E) were quantified also shown. All data expressed as mean±SD. * denotes P<0.05 vs. Sham groups at the same time point and †, P<0.05 vs. I/R group at the same time point.

Since inhibition of SERCA activity could induce ER stress [12], [28], [29], and preservation of SERCA activity could attenuate ER stress during I/R process [12]. Our results demonstrated that SERCA activity was preserved at 3 h of reperfusion, however, was significantly suppressed at 24 h of reperfusion. And this down-regulated SERCA activity was in fact recovered by fasudil infusion (Fig. 3A, the rats studied are the same as in Fig. 2A).

While endogenous phosphorylated PLB level, an active form of regulatory protein for SERCA activity, was up-regulated at both 3 and 24 h of reperfusion (*P*<0.05, I/R group vs. sham controls, at both 3 and 24 h, respectively), fasudil infusion, however, resulted in a further increase at both time points (*P*<0.05, respectively), without any differences between the two time points (*P*>0.05) (Fig. 3B & C, the rats studied are the same as in Fig. 2A).

**Figure 4:**
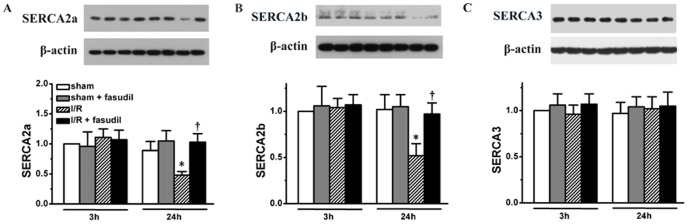
Different SERCA isoform expressions were quantified. Using the same heart tissue described in figure 2, western blotting was performed using specific antibodies to measure the expression levels of different isoforms of SERCA. Panel A showed representative bands of SERCA2a for each group rats with quantification shown in bar graph, Panel B showed quantification of SERCA2b, and Panel C for SERCA3. All data expressed as mean±SD. * denotes P<0.05 vs. Sham groups; †, P<0.05 vs. I/R group at the same time point.

The SERCA2 protein expression in I/R group remained at the same level as in sham controls at 3 h of reperfusion, but was significantly decreased at 24 h of reperfusion, which was recovered by fasudil administration (Fig. 3D&E, the rats studied are the same as in Fig. 2A), suggesting that fasudil improved SERCA activity through increases in both PLB phosphorylation and SERCA2 expression level. We then tested the different expression levels of the two isoforms of SERCA2, including SERCA2a and SERCA 2b during the reperfusion process. In fact, both SERCA2a and SERCA2b exhibited similar pattern of changes as total SERCA2, demonstrating significant decreases in their expression levels at 24 h of reperfusion. In contrast, the expression of total SERCA3 did not show any changes during the first 24 h reperfusion period (Fig. 4, the rats studied are the same as above).

### Both PI3K/Akt and JAK2/STAT3 pathways play differential role in modulating p-PLB and SERCA activity by fasudil

To further elucidate the underlying mechanisms through which fasudil regulates PLB phosphorylation level and SERCA expression and activity, we investigated whether PI3K/Akt, an important component of RISK pathway [3] and JAK2/STAT3, a component of SAFE pathway [4], [30] were involved.

**Figure 5:**
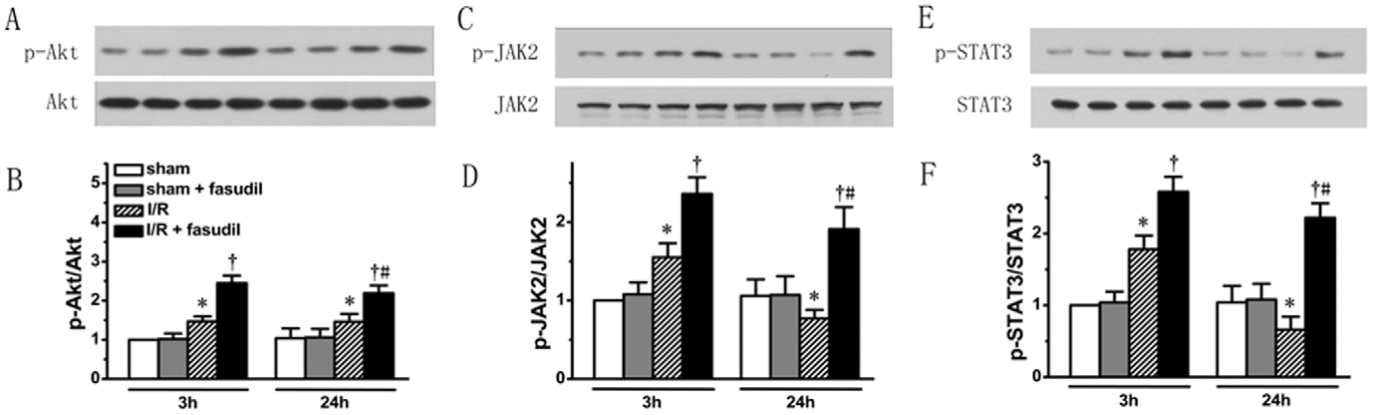
Both PI3K/Akt and JAK2/STAT3 pathway activities were quantified. Using the same heart tissue described in figure 2, western blotting was performed for each group targeting phosphorylation levels of Akt (representative protein bands shown in Panel A and quantified in bar graph in Panel B), phosphorylation levels of JAK2 (Panel C and D) and STAT3 (Panel E and F). All data expressed as mean±SD. * denotes P<0.05 vs. Sham groups; †, P<0.05 vs. I/R group at the same time point and #, P<0.05 vs. I/R+fasudil group at 3 hours of reperfusion.

First, using the same tissue samples obtained above, the activation of these two signaling pathways was measured. We showed that at 3 h in I/R group endogenous Akt, JAK2 and STAT3 phosphorylation levels were up-regulated (*P*<0.05 vs. sham controls, respectively). However, at 24 h of reperfusion the p-JAK2 and p-STAT3 levels were below those of sham controls (*P*<0.05, respectively), whereas Akt remained at the same phosphorylation level as at 3 h of reperfusion. Fasudil infusion resulted in even higher phosphorylation levels of Akt, JAK2 and STAT3 at both 3 h and 24 h of reperfusion (*P*<0.05 vs. I/R group, respectively), although the p-JAK2 and p-STAT3 levels induced by fasudil were significantly less at 24 h than at 3 h of reperfusion (*P*<0.05, respectively) (Fig. 5). Thus, we provide evidence that fasudil can activate both PI3K/Akt and JAK2/STAT3 pathways.

**Figure 6:**
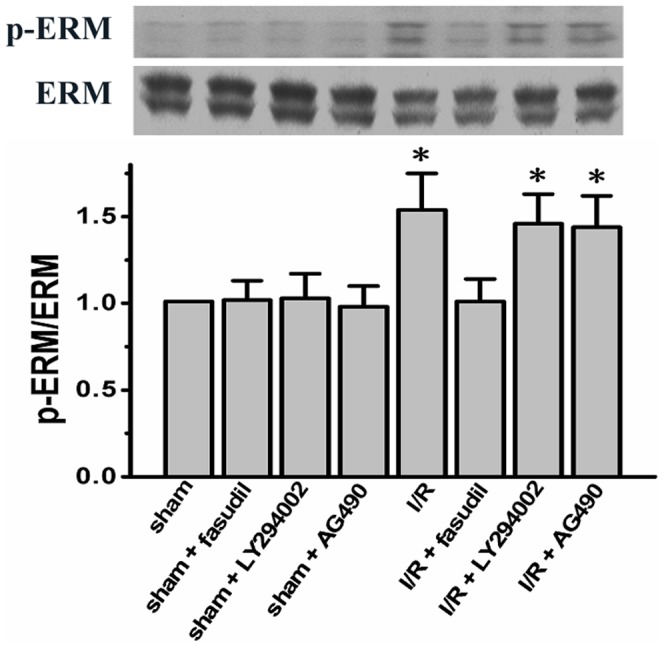
ROCK activity was measured in the presence of either PI3K/Akt inhibitor, LY294002, or JAK2 inhibitor, AG490, by quantifying the phosphorylation level of ERM. Representative bands for p-ERM and total ERM were shown for each group rats (n = 6 for either sham or I/R group rats treated with LY294001 or AG490, the other group rats are the same as shown above). All data expressed as mean±SD. * denotes P<0.05 vs. sham group.

Using specific pharmacological blockers, we went further to test whether activation of the two signaling pathways was involved in regulating ER stress responses via modulating PLB phosphorylation and SERCA protein expression. We showed that both PI3K/Akt inhibitor, LY294002, and JAK2 inhibitor, AG490, did not directly inhibit the ROCK activity as both agents did not change the phosphorylation levels of ERM, a downstream target of ROCK (*P*<0.05. Fig. 6) (n = 6 for either sham or I/R group rats treated with PI3K/Akt or JAK2 inhibitors, the other groups rats are the same as shown above).

**Figure 7:**
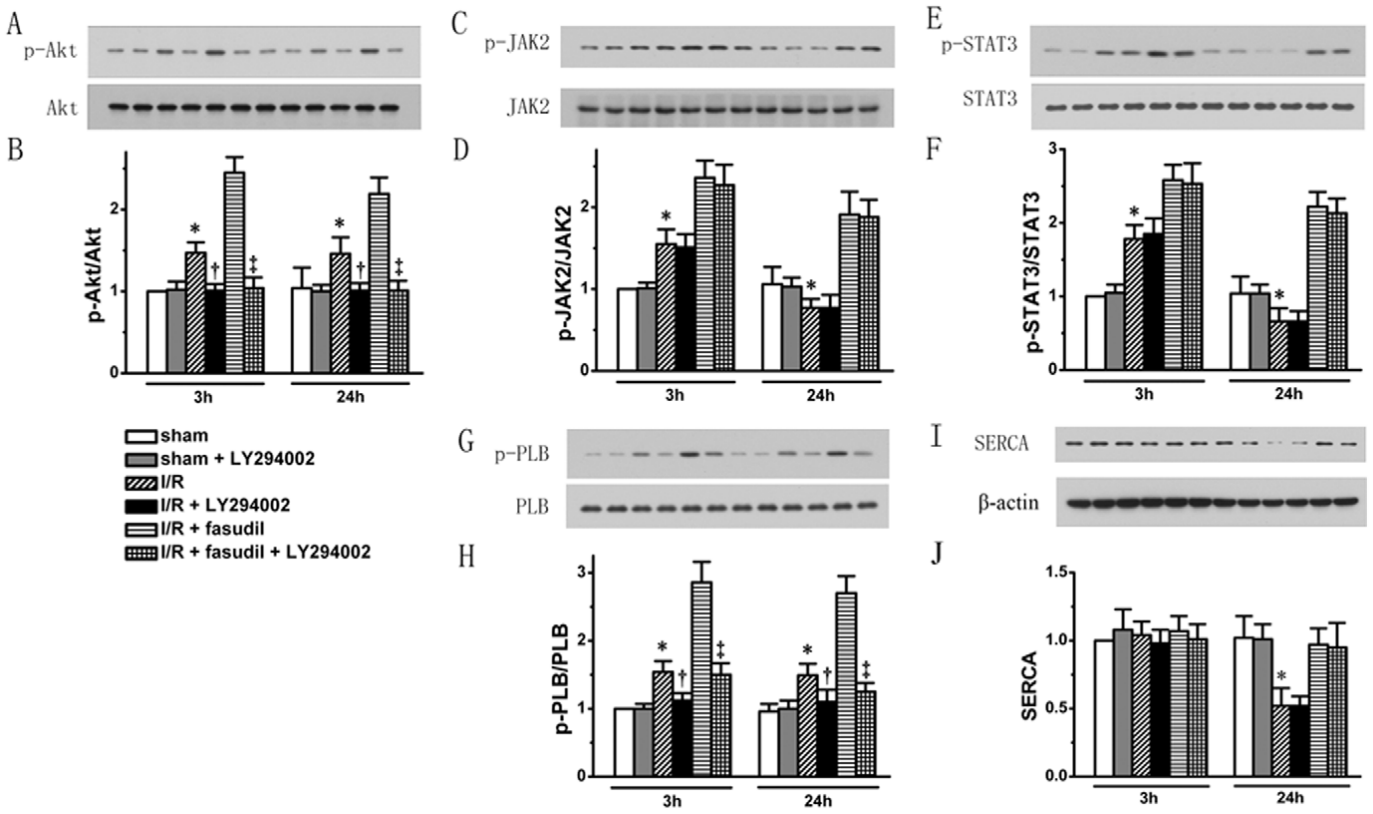
The role of PI3K/Akt signaling in the protection by fasudil was evaluated by using an Akt inhibitor, LY294002. Sham group, I/R group and I/R+fasudil group were either given LY294002 or placebo (n = 6 for each group rats treated with LY294002 for both time points, all the other rats are the same as shown in above). Western blotting was performed, targeting phosphorylation levels of Akt (representative protein bands shown in Panel A and quantified in bar graph in Panel B); JAK2 (Panel C & D), STAT3 (Panel E & F) and PLB (Panel G & H). SERCA protein expression levels (Panel I & J) were also analyzed. All data expressed as mean±SD. * denotes P<0.05 vs. Sham groups; †, P<0.05 vs. I/R group at the same time point and ‡, P<0.05 vs. I/R+fasudil group at the same time point.

Infusion of LY294002 completely abolished both endogenous and fasudil induced Akt phosphorylation, without affecting the levels in sham controls (Fig. 7A&B). Intriguingly, inhibition of PI3K/Akt did not affect the phosphorylation levels of both JAK2 and STAT3 (Fig. 7 C–F), however, completely abolished endogenous PLB phosphorylation shown in I/R groups (*P*<0.05, at 3 and 24 h of reperfusion, respectively), and significantly attenuated PLB phosphorylation levels induced by fasudil at both time points of reperfusion (*P*<0.05, respectively). Meanwhile, LY294002 did not affect the SERCA expression levels (Fig. 7 G–J) (n = 6 for each group rats treated with LY294002, all the other rats studied are the same as shown in above).

**Figure 8:**
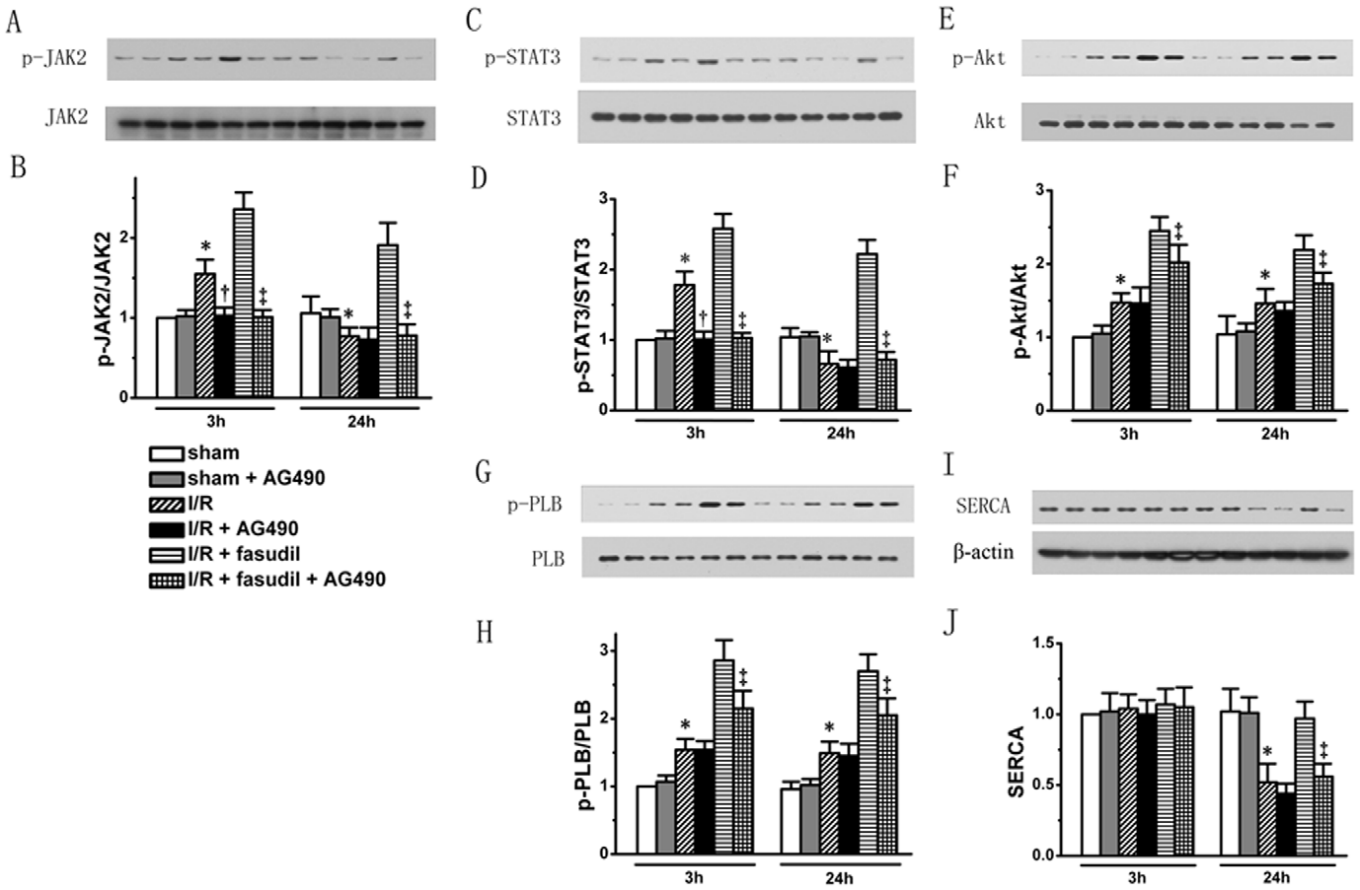
The role for JAK2/STAT3 signaling pathway was also tested by using pharmacological inhibitor. Sham group, I/R group and I/R+fasudil group were either given AG490 or placebo (n = 6 for each group rats treated with AG490 for both time points, all the other rats are the same as shown in above). Western blotting was performed, targeting phosphorylation levels of Akt (Panel A showed representative protein bands for each group with quantification shown in bar graph in Panel B), JAK2 (Panel C & D), STAT3 (Panel E & F) and PLB (Panel G & H). SERCA protein expression levels (Panel I & J) were also analyzed. All data expressed as mean±SD. * denotes P<0.05 vs. Sham groups at the same time point; †, P<0.05 vs. I/R group at the same time point and ‡, P<0.05 vs. I/R+fasudil group at the same time point.

In contrast, JAK2 inhibition not only completely abolished JAK2 and STAT3 phosphorylation (*P*<0.05, respectively) (Fig. 8A–D), but also blocked the increase in Akt phosphorylation level induced by fasudil at both 3 h and 24 h of reperfusion (*P*<0.05, respectively). The endogenous Akt phosphorylation (*P*>0.05, respectively) level was, however, not affected (Fig. 8E&F). The effect of JAK2/STAT3 inhibition was associated with a loss of beneficial effects of fasudil infusion on enhanced PLB phsophorylation (Fig. 8G&H) and improved SERCA protein expression level (Fig. 8I&J) (n = 6 for each group rats treated with AG490, all the other rats studied are the same as shown in above).

### RISK and SAFE pathways exerted their roles in improving ER stress and reducing IS in a time dependent fashion

**Figure 9:**
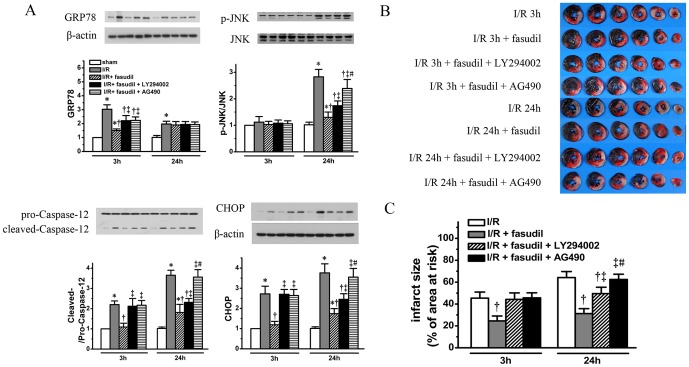
To roles of PI3K/Akt and JAK2/STAT3 signaling in ER stress modulated by fasudil were also evaluated. Using the same heart tissue obtained as described as in Fig. 5 and Fig. 6, ER stress related apoptosis protein markers, including GRP78, phospho-JNK, cleaved Caspase-12 and CHOP were quantified (panel A). IS were measured for I/R rats receiving fasudil with either LY294002 or AG490 at both 3 and 24 hours (n = 8 at each time point per group), the results were compared with I/R group and I/R with fasudil group (using the same data from figure 2). Representative heart slices with TTC staining are shown in panel B with quantification shown in bar graph (Panel C). All data shown are expressed as mean±SD. * denotes P<0.05 vs. sham group at the same time point; †, P<0.05 vs. I/R group at the same time point; ‡, P<0.05 vs. I/R+fasudil group at the same time point and #, P<0.05, vs. I/R+fasudil+LY294002 group at 24 hours of reperfusion.

The PI3K/Akt and JAK2/STAT3 were differentially involved in regulating ER stress dependent apoptosis by fasudil infusion. Both LY294002 and AG490 completely abolished the protection offered by fasudil against ER stress dependent apoptosis signaling at 3 h of reperfusion. In contrast, at 24 h of reperfusion, inhibition of PI3K/Akt signaling only partially blocked the suppression of ER stress dependent apoptosis signaling whereas JAK2/STAT3 inhibition by AG490 resulted in a total loss of this protection by fasudil (Fig. 9A. The samples used are the same as shown in above).

The different degrees of roles for PI3K/Akt and JAK2/STAT3 pathways in modulating ER stress dependent apoptosis activation were also reflected by the changes in IS. Again, both PI3K/Akt and JAK2/STAT3 inhibition abolished the protection against I/R injury at 3 h of reperfusion (IS: I/R+fasudil+LY294002, 44.24±5.9%; I/R+fasudil+AG490, 45.63±4.61%. *P*<0.05, vs. IS: I/R+fasudil group, 24.54±4.52%, respectively). However, at 24 h of reperfusion, PI3K/Akt inhibition had much less effects on reduction in IS by fasudil, only exhibiting a partial reversal in IS (IS: I/R+fasudil+LY294002, 49.54±5.8%, vs. IS: I/R+fasudil group, 31.24±4.55%. *P*<0.05), whereas JAK2/STAT3 inhibition still completely abolished this protection (IS: I/R+fasudil+ AG490, 62.43 ±4.79%; vs. IS: I/R+fasudil, 31.24±4.55%. P<0.05) (Fig. 9B&C) (n = 8 for group rats receiving either LY294002 or AG490 at each time point. The same data for I/R rats without inhibitors as described above were used for comparison).

## Discussion

In the present study we demonstrate dynamic changes in IS, with an abrupt increase in IS from 3 h to 12 h of reperfusion, however, without a further increase at 24 h of reperfusion. We also show that this time course of changes in I/R injury is associated with a stronger ER stress responses at 24 hour reperfusion. These findings are consistent with our previous report [17]. Importantly, our data also show that impaired SERCA activity at 24 h of reperfusion contributes to increased ER stress responses, which results in a time-dependent increase in I/R injury, as shown in IS quantification. Interestingly, ROCK inhibition can significantly improve SERCA activity to attenuate time-course changes in ER stress, resulting in reduced time-related changes in IS. Using pharmacologic blockers, we further demonstrate that activated JAK2/STAT3 signaling induced by fasudil infusion plays major roles in regulating SERCA activity through p-PLB and SERCA expression at 24 h of reperfusion, while PI3K/Akt signaling pathway shares much less roles.

### ROCK inhibition improves dynamic ER stress responses and attenuates time course increase in I/R injury

Our data support the concept that I/R injury is a dynamic pathological process [14], [15], [16] and that ER stress responses are involved in the time course of changes in IS. We have shown that both IS and ER stress related apoptosis signaling pathways exhibited abrupt increases at 24 h of reperfusion compared with at 3 h of reperfusion. Thus, the pattern of changes in IS and expression of ER stress related apoptosis signaling including CHOP, cleaved Caspase-12 and p-JNK is similar to that observed in our previous report where I/R injury was induced by 30 min ischemia instead and measured at both 2 and 24 h of reperfusion. We noticed 45 min ischemia results in a higher IS at both early (i.e. 2–4 h of reperfusion) and late (24 h of reperfusion) phase of reperfusion in the present study. Importantly, ER stress dependent apoptosis is also associated with significant increases in percentage of TUNEL staining positive cells and caspase-3 activity, which account for the temporal increase in IS. Of note, ROCK inhibition can significantly attenuated the time dependent activation of ER stress dependent apoptosis signaling.

ER stress has been shown to be involved in various pathological processes [31], including cardiovascular diseases [32]. However, prolonged and severe ER stress eventually triggers ER stress related apoptosis signaling [9] including activation of CHOP, Caspase-12 and JNK signaling pathways, leading to I/R injury [28], [33]. Interestingly, in our study these three pathways exhibit a similar pattern of activation, with a peak present at the late phase of reperfusion (i.e. 12 h after reperfusion). This is in line with our previous study where dynamic ER stress also reached its peak at late phase of reperfusion following a 30 min ischemia period. This also indicates that similar ER stress activation process when ischemia time varied between 30 and 45 min, yet a longer ischemia event contributing to an increase in I/R injury at late phase of reperfusion. Our data is also quite different from a previous report where a more mild chronic pathologic process activated only CHOP expression without involving JNK and Caspase-12 pathways [34]. It will be interestingly to look at whether different duration of ischemia would result in a different pattern of ER stress activation; a further study is certainly warranted.

To exclude the possibility that any off-target effects might be involved in the protective effects of fasudil, we also tested the effects of Y-27632, another ROCK inhibitor that is structurally different from fasudil. We showed that Y-27632 can also modulate ER stress and significantly decreased dynamic changes in I/R injury. More importantly, Y-27632 protected against I/R injury not only at 3 h of reperfusion when ER stress plays much less role in I/R injury, but also at late phase (i.e. 24 h) of reperfusion when temporal increase in I/R injury occurs. This is strikingly in contrast to the effects of TUDCA which only showed its effect against I/R injury at 24 h of reperfusion at which similar effects were observed between Y-27632 and high dose of TUDCA. Based on the data presented in our study, we reason that at early reperfusion, ER stress does not play a major role in I/R injury. Instead, activation of ER stress contributes to temporal changes in I/R injury. On the other hand, at early reperfusion injury, the protection offered by ROCK inhibition is probably due to its biological effects beyond modulating ER stress.

### Mechanisms underlying the protection against ER stress responses by ROCK inhibition

RhoA, a small GTPase protein, has been shown to play an essential role in a variety of cardiovascular diseases [35], [36]. Inhibition of ROCK, a downstream target of RhoA, results in a significant reduction in IS [18], [27]. Our data further provide the evidence that ROCK inhibition can reduce the IS via attenuation of ER stress dependent apoptosis signalings. Intriguingly, fasudil results in a decrease in GRP78 expression at 3 h of reperfusion, without having further effects on its expression at 24 hours of reperfusion. The strikingly different responses to fasudil infusion at two time points of reperfusion may be related to different stage of pathologic process, or therapeutic strategy [7], [37]. While enhanced chaperone expression has been shown to attenuate ER stress [38], [39], [40], our data showed that improved ER stress is associated with lower chaperone expression which is consistent with a previous study where ischemic preconditioning resulted in lower expression of chaperone calreticulin, possibly through enhanced expression of p38MAPK [37]. In addition, we showed that administration of chemical chaperone TUDCA can actually suppressed endogenous increases in GRP78 and CHOP expression, which was also associated with reduction of temporal increase in I/R injury. Thus, our data not only further supports the role of ER stress in dynamic changes in IS at 24 h of reperfusion, but also suggests different approach to modulating ER stress might have different reactions. Thus, our data suggests that the increase in spontaneous GRP78 expression reflects an endogenous response during I/R injury process while the reduced chaperone expression after fasudil infusion is probably secondary to improved ER stress by ROCK inhibition. On the other hand, ROCK inhibition might not act through enhanced chaperone expression.

It is well established that Ca^2+^ homeostasis is critical in maintaining normal ER function and impaired Ca^2+^ handling has been shown to be involved in myocardial I/R injury [41], [42], [43] for which SERCA activity plays a key role [44], [45]. In our study, SERCA activity was impaired only at 24 hours of reperfusion due to the down-regulated SERCA protein expression level. Surprisingly, at early phase of reperfusion even though the SERCA protein expression and activity did not show any changes, fasudil infusion results in an increase in phosphorylation level of PLB, which has been considered to enhance SERCA activity [46]. The reason for this is still not fully understood in the present study. We reason that polyol pathway could potentially have impaired SERCA activity without affecting the protein expression levels which compromised the effect of enhanced PLB phosphorylation levels. Importantly, our study demonstrates that impaired SERCA activity contributed to the time related ER stress dependent apoptosis signalings, which can be rescued by ROCK inhibition via increased SERCA expression as well as enhanced PLB phosphorylation levels.

### Differential roles for PI3K/Akt and JAK2/STAT3 pathways in regulating SERCA activities by ROCK inhibition

It has been shown that PI3K/Akt signaling as a key component of RISK pathway [3] is involved in cardiac protection provided by ROCK inhibition [18], [27]. In the present study, we confirm that PI3K/Akt is involved in modulating ER stress during reperfusion which has been shown before [17], [47]. But, we further show that activation JAK2/STAT3 pathway, a component of SAFE pathway [48] is also involved in the protection offered by fasudil infusion. More importantly, we demonstrate that PI3K/Akt and JAK2/STAT3 pathways are actually differentially involved in mediating SERCA activity. However, at late phase of reperfusion the SERCA activity is impaired, probably due to either accumulated effects of protein oxidation [49], [50] or down-regulated transcription activity [51]. At this time point, SERCA activity was mainly regulated by JAK2/STAT3 signaling pathway. Thus, we demonstrated an important role of JAK2/STAT3 signaling in maintaining SERCA protein and activity in a time dependent fashion. This is also consistent with the fact that JAK2/STAT3 inhibitor can almost abolished the protective effects by ROCK inhibitor at late phase of reperfusion, whereas the PI3K/Akt inhibitor did not completely abolished this protection.

### Limitations

Our data only showed that impaired SERCA2 activity during reperfusion can be enhanced by ROCK inhibition, and this improved SERCA activity was associated with JAK2/STAT3 activation, however, we were not able to show how JAK2/STAT3 activation up-regulates SERCA2 expression. Further study is needed to further explore the mechanisms involved for mediating SERCA2 expression or activities. In addition, we showed a dynamic change in SERCA2 expression and activity during I/R injury process. The expressions of the two isozymes of SERCA2, SERCA2a and SERCA2b, and total SERCA3 have also been tested. Great progress has been made in understanding the biology of different isoforms of SERCA2 and SERCA3 [52]. It has been shown that SERCA2a is the main isoform present in the heart, whereas SERCA2b, a ubiquitously expressed isozyme, shows much lower level in the heart [53]. Although study showed that SERCA2b could be induced in the setting of pathological process, such as ER stress [29], however, the function of SERCA2a may not be compensated or replaced [54]. In the present study, we have shown that during heart I/R injury process, SERCA2a and SERCA2b exhibited similar pattern of changes. In addition, using pan-SERCA3 antibody we did not observe change in its expression, however, we cannot exclude interplay between different isoforms of SERCA3. A further study is certainly warranted to explore this interesting research area. Of note, even though our previous study showed that the cardiomyocytes are the major cells that showed positive immuno-staining for both GRP78 and CHOP that were detected by con-focal microscope, in the present study we still could not quantify to what extent the other cells including leukocytes contribute to ER stress signaling, due to technical difficulties. Nevertheless, we still showed that ER stress activation caused the time course changes in I/R injury which can be reversed by enhancing SERCA activity with ROCK inhibition.

### Conclusion

In the present study we have demonstrated that impaired SERCA activity causes ER stress dependent apoptosis, leading to time-related increase in I/R injury. ROCK inhibition by fasudil can improve SERCA activity, which results in significant decreases in ER stress responses and attenuated temporal increase in I/R injury. Moreover, the activated JAK2/STAT3 signaling pathway plays a major role in modulating SERCA activity, where PI3K/Akt shares comparatively less role at the late phase of reperfusion. Further study is warranted to investigate the molecular mechanisms by which the two different signaling pathways regulate SERCA activity during I/R injury process.

## Supporting Information

Figure S1 The effects of chemical chaperone, TUDCA, and another ROCK inhibitor, Y-27632, on temporal changes in I/R injury were measured. Representative sequential LV slices were also obtained from different dose TUDCA groups (100, 200 and 400 mg/Kg groups) and Y-27632 group rats (n = 8 for each time point/group), the rats for I/R group are the same as shown in Fig. 1. IS was quantified by combination of Evans blue and TTC staining using the same method as shown in Fig. 1 and the bar graph at the lower panel shows the percentage of LV weight of infarct area over LV weight at area at risk. All the data presented in the bar graph were expressed as mean±SD. * denotes P<0.05 vs. I/R group; †, P<0.05 vs. I/R rats treated with TUDCA at 400 mg/Kg at each time point, respectively.
(TIF)

Figure S2 The effects of chemical chaperone, TUDCA, and ROCK inhibitor, Y-27632, on time course changes in ER stress were also measured. GRP78 and CHOP expression levels were also quantified by Western Blot to assess ER stress at both 3 h(upper panels) and 24 h(lower panels) of reperfusion with representative bands shown in the left panel, and quantified in the corresponding bar graphs, respectively. All data expressed as mean±SD. * denotes P<0.05 vs. Sham groups; †, P<0.05 vs. I/R group; ‡, P<0.05 vs. I/R group rats treated with TUDCA at 100 mg/Kg at the same time point respectively.
(TIF)
