# Supplementary figures and images for: Fasudil Protects the Heart against Ischemia-Reperfusion Injury by Attenuating Endoplasmic Reticulum Stress and Modulating SERCA Activity: The Differential Role for PI3K/Akt and JAK2/STAT3 Signaling Pathways
Source: PLoS One. 2012 Oct 31;7(10):e48115. doi: 10.1371/journal.pone.0048115 (PMC3485283; doi:10.1371/journal.pone.0048115)

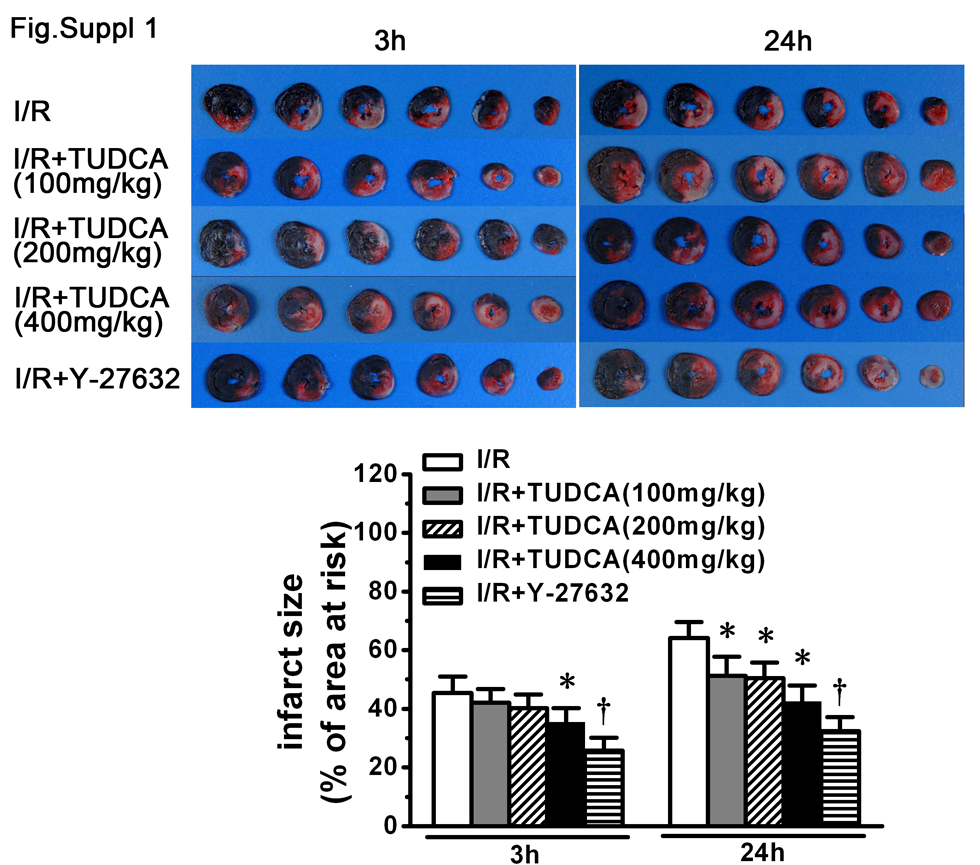

Supplement: Figure S1 — The effects of chemical chaperone, TUDCA, and another ROCK inhibitor, Y-27632, on temporal changes in I/R injury were measured. Representative sequential LV slices were also obtained from different dose TUDCA groups (100, 200 and 400 mg/Kg groups) and Y-27632 group rats (n = 8 for each time point/group), the rats for I/R group are the same as shown in Fig. 1. IS was quantified by combination of Evans blue and TTC staining using the same method as shown in Fig. 1 and the bar graph at the lower panel shows the percentage of LV weight of infarct area over LV weight at area at risk. All the data presented in the bar graph were expressed as mean±SD. * denotes P<0.05 vs. I/R group; †, P<0.05 vs. I/R rats treated with TUDCA at 400 mg/Kg at each time point, respectively. (TIF) [file pone.0048115.s001.tif]

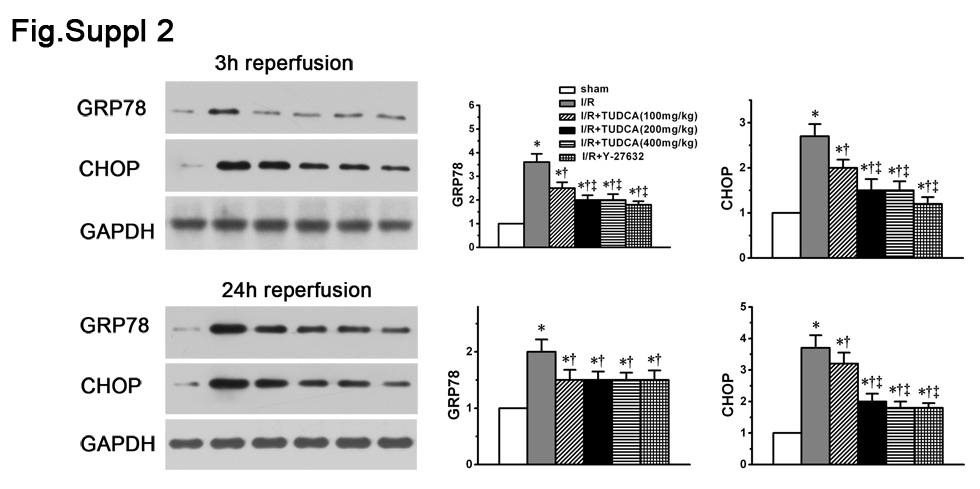

Supplement: Figure S2 — The effects of chemical chaperone, TUDCA, and ROCK inhibitor, Y-27632, on time course changes in ER stress were also measured. GRP78 and CHOP expression levels were also quantified by Western Blot to assess ER stress at both 3 h(upper panels) and 24 h(lower panels) of reperfusion with representative bands shown in the left panel, and quantified in the corresponding bar graphs, respectively. All data expressed as mean±SD. * denotes P<0.05 vs. Sham groups; †, P<0.05 vs. I/R group; ‡, P<0.05 vs. I/R group rats treated with TUDCA at 100 mg/Kg at the same time point respectively. (TIF) [file pone.0048115.s002.tif]
